# Genomic epidemiology and antimicrobial resistance of *Corynebacterium macclintockiae*, the predominant species of human pathogens within the *Corynebacterium jeikeium* complex

**DOI:** 10.1128/jcm.00500-25

**Published:** 2025-07-24

**Authors:** Sohei Harada, Kohji Komori, Kenya Yukawa, Brian Hayama, Kazumi Takehana, Kageto Yamada, Asako Doi, Tomoo Saga, Masakazu Sasaki, Yoshiro Hadano, Masahiro Suzuki, Kyoko Yokota, Jun Suzuki, Koki Kikuchi, Yohei Doi, Kazuhiro Tateda

**Affiliations:** 1 Department of Microbiology and Infectious Diseases, Toho University School of Medicine, Ota-ku, Tokyo, Japan; 2 Department of Microbiology and Infectious Diseases, Toho University Graduate School of Medicine, Ota-ku, Tokyo, Japan; 3 Department of Infectious Diseases, Cancer Institute Hospital, Japanese Foundation for Cancer Research 13609, Koto-ku, Tokyo, Japan; 4 Clinical Laboratories, Cancer Institute Hospital, Japanese Foundation for Cancer Research 13609, Koto-ku, Tokyo, Japan; 5 Department of Infectious Diseases, Kobe City Medical Center General Hospital 26330, Kobe, Hyogo, Japan; 6 Division of Infection Control and Prevention, Akita University Hospital 91996, Akita, Akita, Japan; 7 Department of Clinical Laboratories, Toho University Omori Medical Center 36592, Ota-ku, Tokyo, Japan; 8 Division of Infection Control and Prevention, Shimane University Hospital 653239, Izumo, Shimane, Japan; 9 Department of Microbiology, Fujita Health University School of Medicine 89305, Toyoake, Aichi, Japan; 10 Center for Infectious Disease Education, Kagawa University Hospital 469524, Kida-gun, Kagawa, Japan; 11 Division of Infectious Diseases, Gifu Prefectural General Medical Center 68266, Gifu, Gifu, Japan; 12 Department of Infectious Diseases, Teine Keijinkai Hospital 37009, Sapporo, Hokkaido, Japan; 13 Division of Infectious Diseases, University of Pittsburgh School of Medicine 12317 , Pittsburgh, Pennsylvania, USA; Cleveland Clinic, Cleveland, Ohio, USA

**Keywords:** whole-genome sequencing, *Corynebacterium macclintockiae*, *Corynebacterium jeikeium*

## Abstract

Genomic characteristics and optimal treatment of *Corynebacterium jeikeium* remain largely unknown. We collected clinical information and performed whole-genome sequencing analysis of the causative strains of six cases of *C. jeikeium* infection at a single hospital over a 9-year period. Additionally, whole-genome sequencing analysis was performed on 33 *C*. *jeikeium* strains from cases of bloodstream infection at eight hospitals. Antimicrobial susceptibility testing was performed, and the results were compared to the resistance genes identified. Publicly available genome data of strains of *C. jeikeium* complex, consisting of *C. jeikeium sensu stricto*, *Corynebacterium macclintockiae*, and *Corynebacterium evansiae*, worldwide, were combined with the data from this study to determine the distribution of genomic species. In the single-center study, cases of prosthetic osteoarticular infection, postoperative intra-abdominal infection, and catheter-related bloodstream infection were identified, and the causative strains were genomically identified as *C. macclintockiae*. All but one isolate (32/33, 97.0%) in the eight-center study identified as *C. jeikeium* by matrix-assisted laser desorption ionization-time of flight mass spectrometry were also genomically identified as *C. macclintockiae*. Nosocomial transmission was suggested in three strain pairs by core-genome single nucleotide polymorphism analysis. *C. macclintockiae* strains were generally multidrug-resistant, but all anti-methicillin-resistant *Staphylococcus aureus* agents, including teicoplanin, had favorable activity, and the strains without the *tet*(W) gene (22/38, 57.9%) were susceptible to tetracyclines. Genome analysis of 66 *C*. *jeikeium* complex strains collected worldwide, consisting mainly of clinical strains, re-identified 51 strains (77.3%) as *C. macclintockiae*. This study demonstrates that *C. macclintockiae* is the major genomic species of the *C. jeikeium* complex causing human infections.

**IMPORTANCE:**

Recent widespread use of matrix-assisted laser desorption ionization-time of flight mass spectrometry (MALDI-TOF MS) has facilitated the identification of *Corynebacterium* spp. in microbiology laboratories, thereby raising awareness of the clinical importance of these organisms. Nevertheless, the accumulation of information on genomic characteristics of *Corynebacterium jeikeium* has been significantly limited compared to other pathogenic organisms thus far. In this study, we analyzed causative strains of infections identified as *C. jeikeium* by MALDI-TOF MS, collected from multiple institutions throughout Japan, and found that most of these strains were genomically identified as *Corynebacterium macclintockiae*, a species that has been newly described recently. Collection of clinical information on selected cases showed that *C. macclintockiae* indeed caused invasive infections that required intravenous or long-term oral antimicrobial therapy. Additional analyses using genomic data of *C. jeikeium* complex strains registered in public databases suggest that *C. macclintockiae* is of global clinical importance.

## INTRODUCTION

*Corynebacterium* spp. are catalase-positive gram-positive bacilli, which historically had all been considered to be of low virulence for human infection, exclusive of diphtheria toxin producers (*Corynebacterium diphtheriae*, *Corynebacterium ulcerans*, etc.) (1). However, *Corynebacterium jeikeium*, along with *Corynebacterium striatum*, is known to cause human infections more frequently than other non-diphtheriae *Corynebacterium* spp. In a recent study evaluating the clinical significance of *Corynebacterium* spp. detected from blood cultures, 57 of 81 cases (70.4%) were diagnosed as true bacteremia for *C. jeikeium* or *C. striatum*, identified with RapID CB Plus (Kyokuto Pharmaceutical Industrial Co., Ltd., Tokyo, Japan) or MALDI Biotyper (Bruker Daltonics, Bremen, Germany), compared with only 3 of 34 cases (8.8%) for other species (2). In addition, the recent widespread adoption of matrix-assisted laser desorption ionization-time of flight mass spectrometry (MALDI-TOF MS) has increased the accuracy of identification of *Corynebacterium* spp. in microbiology laboratories, thereby raising awareness of the clinical importance of these organisms (1, 3).

It has been recognized that patients with hematologic malignancies, especially those with neutropenia, are particularly susceptible to *C. jeikeium* infections (4). A recent study also showed that *C. jeikeium* bacteremia occurred predominantly in patients with hematologic malignancies, whereas *C. striatum* bacteremia occurred in patients with diverse underlying diseases (2). *C. jeikeium* is generally multidrug-resistant, and vancomycin has been used as the standard treatment for infections (1, 2, 4).

Information on the genomic characteristics of *C. jeikeium* is scarce. As of 29 December 2024, only 23 whole-genome sequencing data of *C. jeikeium* complex had been deposited in GenBank, which is significantly less than other pathogenic organisms. As a result, the accuracy of the species identification results by MALDI-TOF MS has not been verified using identification with whole-genome sequencing analysis as the gold standard. In addition, there is limited information on the association between resistance gene carriage and antimicrobial susceptibility in *C. jeikeium*. The validity of the current antimicrobial susceptibility breakpoints has also not been sufficiently evaluated.

To fill these knowledge gaps, we collected bacterial isolates detected as causative organisms of invasive infections and identified as *C. jeikeium* by MALDI-TOF MS from multiple hospitals. Information on clinical features, treatment, and prognosis was also collected for cases from one hospital. Genetic characteristics were examined by whole-genome sequencing analysis, and the accuracy of identification by MALDI-TOF MS was verified. Antimicrobial susceptibility testing was also performed extensively. In addition, genomic analysis of *C. jeikeium* complex was performed with additional data from public databases to verify the epidemiological and clinical importance of *Corynebacterium macclintockiae*, a genomospecies of *C. jeikeium* complex.

## MATERIALS AND METHODS

### Single-center study of *C. jeikeium* infections

Bacterial strains determined as the causative organisms of *Corynebacterium* infections by infectious disease specialists and frozen-stored at one hospital (Hospital A) from January 2015 to July 2024 were the candidate strains. Six of these strains identified as *C. jeikeium* using the MALDI Biotyper with BDAL library v11 at the central facility (Toho University) with a score value of 2.000 or more were finally included. BDAL library v11 contained data of mass spectra for 11 *C*. *jeikeium* reference strains, but no data for *C. macclintockiae* and *Corynebacterium evansiae*.

The following clinical information of the six patients was collected from electronic medical records: age; sex; setting in which the infection occurred (5); Charlson Comorbidity Index (6); immunocompromising conditions (Table S1); diagnosis of infection; Pitt bacteremia score (7); bacterial culture testing results; antimicrobial treatment targeting *C. jeikeium* until Day 90; clinical improvement by Day 14 defined as a composite of improvement of fever and local inflammatory findings, and documented negative cultures; and mortality by Day 90. Day 0 was defined as the day when the culture specimen with *Corynebacterium* growth was collected.

Whole-genome sequencing analysis of *C. jeikeium* isolates was performed with MiSeq (Illumina, San Diego, CA, USA). DNA was extracted from bacterial cells using the bead-beating method with EZ-Beads (Promega K.K., Tokyo, Japan), followed by processing with a combination of magLEAD 6gC and MagDEA Dx SV with PS protocol (Precision System Science Co., Ltd., Chiba, Japan). DNA libraries were prepared using Illumina DNA Prep (Illumina, San Diego, CA, USA) and sequenced with MiSeq with 300 bp paired-end reads. Raw reads generated by MiSeq were trimmed for adapter sequences and those not passing a quality score of Q30 or higher using the Trimmomatic tool (version 0.39) with the following parameters: LEADING:30, TRAILING:30, SLIDINGWINDOW:4:15, MINLEN:100, and HEADCROP:5. Trimmed reads were assembled using SPAdes (version 3.15.4) with paired-end reads.

The average nucleotide identity (ANI) of the obtained draft genome and genomes of reference strains, *C. jeikeium* NCTC 11915^T^ (GenBank accession number: NC_007164), *C. macclintockiae* c9Ua_112^T^ (GenBank accession number: NZ_JAKMUV010000000), and *C. evansiae* c8Ua_174^T^ (GenBank accession number: NZ_JAKMUT010000000), was calculated using FastANI (version 1.33), and genetic identification of the bacterial species was performed using an ANI threshold of 95% or higher (8). Hereafter, *C. jeikeium*, *C. macclintockiae*, and *C. evansiae* are collectively referred to as *C. jeikeium* complex when necessary.

### Eight-center study of bacteremic strains of *C. jeikeium* complex

**Fig 1:**
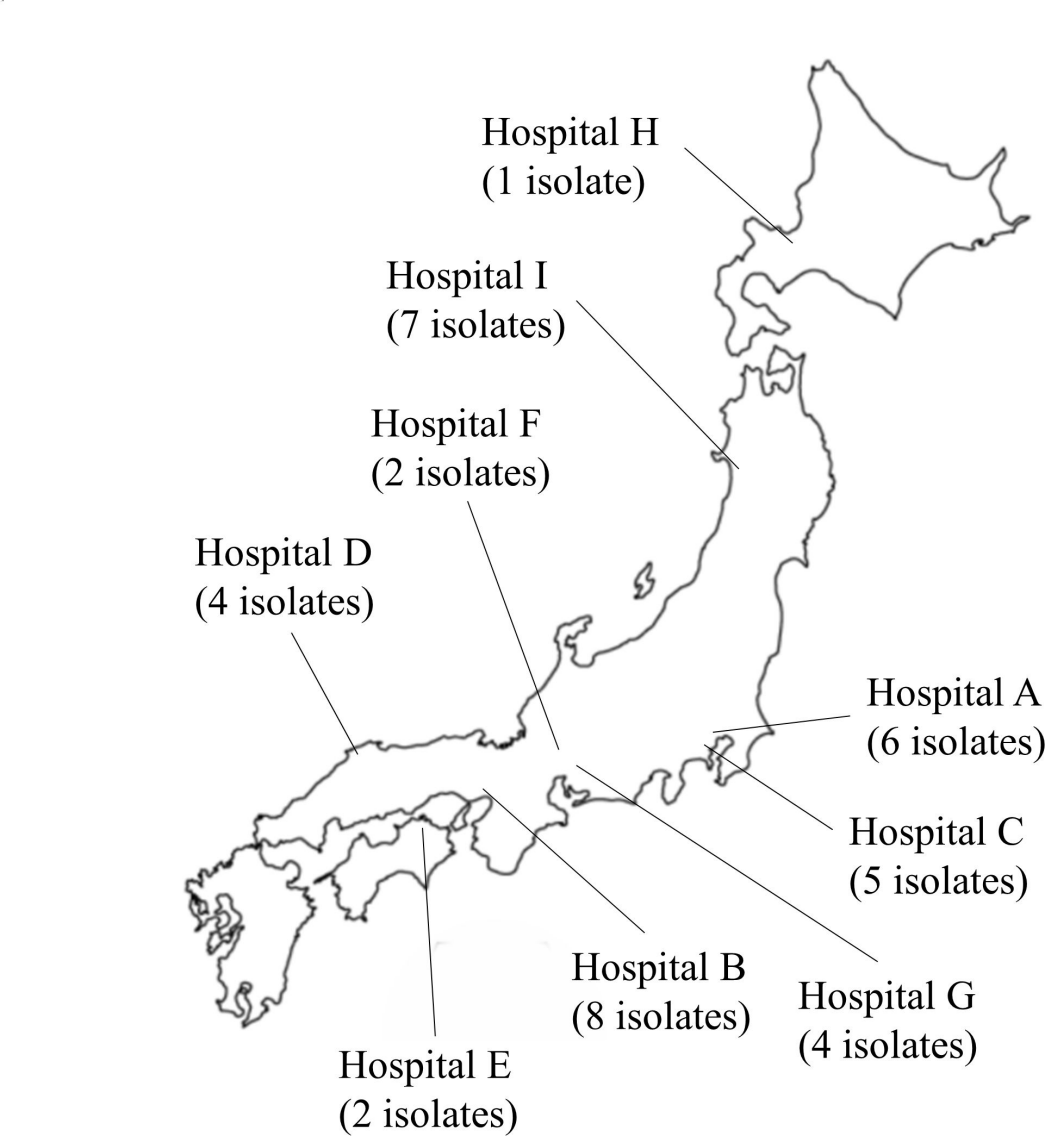
Geographic location of participating facilities and number of strains collected in this study.

Thirty-three strains detected from multiple sets of blood culture and identified as *C. jeikeium* by MALDI-TOF MS were collected from eight facilities in Japan (Hospitals B to I) (Fig. 1). The strains were re-identified using MALDI Biotyper at the central facility, and whole-genome sequencing analysis was performed with the same protocol as the single-center study.

### Antimicrobial susceptibility testing of *C. jeikeium* complex strains

Antimicrobial susceptibility testing was performed by the broth microdilution method for a total of 39 *C. jeikeium* complex strains collected through the single-center study and the eight-center study. Minimum inhibitory concentrations (MICs) were determined by the broth microdilution method and interpreted according to CLSI M45-ED3 guidelines (9). Cation-adjusted Mueller-Hinton broth supplemented with lysed horse blood was used, and calcium concentration was adjusted to 50 µg/mL in the testing of daptomycin susceptibility. MICs were determined for penicillin (Sigma-Aldrich Co. LLC, St. Louis, MO, USA) (range: 0.06–64 µg/mL), ampicillin (Fujifilm Wako Pure Chemical Corporation, Osaka, Japan) (0.06–64 µg/mL), ampicillin/sulbactam (Fujifilm Wako Pure Chemical Corporation) (0.06/0.03 to 64/32 µg/mL), ceftriaxone (Sigma-Aldrich Co. LLC) (0.12–16 µg/mL), cefepime (United States Pharmacopeia, Rockville, MD, USA) (0.12–16 µg/mL), meropenem (Fujifilm Wako Pure Chemical Corporation) (0.06–8 µg/mL), levofloxacin (LKT Laboratories, Inc., St. Paul, MN, USA) (0.03–64 µg/mL), ciprofloxacin (LKT Laboratories, Inc.) (0.03–64 µg/mL), tetracycline (Sigma-Aldrich Co. LLC) (0.03–64 µg/mL), doxycycline (Tokyo Chemical Industry Co., Ltd., Tokyo, Japan) (0.03–64 µg/mL), minocycline (Tokyo Chemical Industry Co., Ltd.) (0.03–64 µg/mL), erythromycin (Fujifilm Wako Pure Chemical Corporation) (0.12–16 µg/mL), clindamycin (Tokyo Chemical Industry Co., Ltd.) (0.12–16 µg/mL), gentamicin (Fujifilm Wako Pure Chemical Corporation) (0.25–32 µg/mL), trimethoprim (Fujifilm Wako Pure Chemical Corporation)/sulfamethoxazole (Shionogi & Co., Ltd., Osaka, Japan) (2.3/0.12 to 152/8 µg/mL), vancomycin (Sigma-Aldrich Co. LLC) (0.12–16 µg/mL), teicoplanin (Aventis Pharma, Tokyo, Japan) (0.12–16 µg/mL), daptomycin (Tokyo Chemical Industry Co., Ltd.) (0.03–4 µg/mL), and linezolid (Tokyo Chemical Industry Co., Ltd.) (0.06–8 µg/mL). For teicoplanin, levofloxacin, and minocycline, for which breakpoints are not set in CLSI M45, breakpoints of vancomycin, ciprofloxacin, and doxycycline were used, respectively. *Streptococcus pneumoniae* ATCC 49619, *Escherichia coli* ATCC 25922 for gentamicin and minocycline, and *E. coli* ATCC 35218 for ampicillin-sulbactam were used as quality control strains.

### Molecular characterization of *C. jeikeium* complex strains

Antimicrobial resistance genes of *C. jeikeium* complex strains were identified using ResFinder (version 4.6.0) (http://genepi.food.dtu.dk/resfinder). In addition, the class A β-lactamase gene (*bla*_Coryne-A_), which has been documented across multiple *Corynebacterium* species in previous studies, was identified manually using Basic Local Alignment Search Tool (https://blast.ncbi.nlm.nih.gov/Blast.cgi) (10). Mutations in the quinolone resistance-determining regions (QRDRs) of *gyrA* were also identified using the nucleotide sequence of *gyrA* in *C. jeikeium* ATCC43734 strain (GenBank accession number: ACYW01000075) as the reference.

Genomic similarity of *C. macclintockiae* isolates was assessed by core-genome single nucleotide polymorphism (SNP) analysis. TUM24530, isolated from a blood culture in the single-center study, was selected as the reference strain and subjected to complete genome analysis using MinION Mk1B (Oxford Nanopore Technologies, Oxford, UK) long-read sequencer. DNA extraction was performed with the protocol used for MiSeq sequencing, and library preparation and two-dimensional sequencing were performed with Rapid Barcoding Kit SQK-RBK114.24 (Oxford Nanopore Technologies) and Flongle flow cell R10.4.1 (Oxford Nanopore Technologies), respectively. Basecalling and demultiplexing were executed by Guppy (version 6.5.7). Flongle reads were adapter-trimmed and quality-filtered using NanoFilt (version 2.6.0) with a quality score cutoff of Q6 and a minimum length of 5,000 bp (11). The generated Flongle reads were assembled using Unicycler (version 0.4.8-beta) in bold mode, in combination with the MiSeq reads trimmed as described above (12). The contigs were polished three times using Pilon version 1.23 based on the MiSeq reads (13).

Core-genome SNP-based phylogenetic analysis was performed using MiSeq reads data of the 38 *C*. *macclintockiae* strains. The reads were aligned to the genome sequences of the reference strain, TUM24530, using the Burrows-Wheeler Aligner with the “SW” algorithm (14). Core-genome alignments were extracted by SAMtools (version 1.21) mpileup with base alignment quality adjustment disabled and reference genome specified explicitly. SNPs and consensus sequences were called using VarScan (version 2.3.9) mpileup2cns with default parameters (15, 16). A temporal phylogenetic tree was constructed based on the maximum likelihood method using PhyML (version 3.3.20241207) with the general time reversible (GTR) substitution model and 100 bootstrap replicates (17). This tree was used as the initial input for ClonalFrameML (version 1.12) to infer homologous recombination events that introduced DNA fragments from outside the phylogenetic cluster, thereby generating a recombination-corrected clonal phylogeny (18). A phylogenetic tree based on core-genome SNPs, excluding homologous recombination regions inferred by ClonalFrameML, was reconstructed using the maximum likelihood method implemented in RAxML (version 8.2.12) with the GTR substitution model and 1,000 bootstrap replicates (19). All trees were visualized using Figtree (version 1.4.4). The number of SNPs in the core genome was calculated using Snp-dists (version 0.7.0; https://github.com/tseemann/snp-dists). For the isolates belonging to the four clusters with high genomic similarity identified in the first analysis, the core-genome SNP analysis was performed again, restricting the analysis to the isolates belonging to each cluster. To estimate the frequency of occurrence of mutations in the genome of *C. macclintockiae*, we additionally performed a whole-genome sequencing of a *C. macclintockiae* isolate (TUM25035) (GenBank accession number: JBLYWG000000000) detected in blood culture about 10 months earlier from the patient from whom one of the study strains (TUM25036) was derived.

### Comparative genomic analysis of *C. jeikeium* complex using registered genomes

All genomic data of *C. jeikeium*, *C. macclintockiae*, and *C. evansiae* registered in GenBank as of 29 December 2024 were downloaded for use in the comparative genomic analysis. Bacterial species of the registered genome data were re-identified by ANI using the genomes of the reference strains of *C. jeikeium*, *C. macclintockiae*, and *C. evansiae*. Core-genome SNP analysis was performed using both publicly available genomic data and those newly acquired in this study. Core-genome SNP analysis was performed using the same method as for the *C. macclintockiae* strains in Japan, except that the exclusion of homologous recombination regions, which would make the core-genome region extremely small (120 bp), was omitted.

In addition, a comparison of the 16S rRNA and *rpoB* sequences of two strains collected in France in a previous study (strains 25850 and 59614) with that of the *C. jeikeium* complex strains with genome data registered in GenBank was performed. The two strains were among 24 clinical strains with high 16S rRNA and *rpoB* sequence similarity to the type strain of *C. jeikeium* collected in France over a 5-year period until 2003, showing a relatively low *rpoB* sequence similarity of 91%–93% compared to other strains (95%–97%) (20). Using the 16S rRNA (GenBank accession number: AY581881) and *rpoB* (GenBank accession number: AY581873) sequences of strain 25850 as reference sequences, phylogenetic analysis with strain 59614 and strains with GenBank-registered genome data was performed. SNP extraction and generation of the phylogenetic tree were performed using the same methods used in the core-genome SNP analysis of the *C. jeikeium* complex.

## RESULTS

### Single-center study of *C. jeikeium* infections

Of the six *C. jeikeium* strains from Hospital A (Table S2), three caused postoperative intra-abdominal infections, and one each caused central venous access port-related bloodstream infection, vertebral osteomyelitis after spine instrumentation surgery, and prosthetic joint infection after knee arthroplasty (Table 1). One case was a healthcare-associated infection, and the remaining cases were hospital-acquired infections. Since Hospital A is a cancer center, all patients had solid tumors, and three also had metastatic tumors. All but one patient had a history of surgery within 30 days and presented with surgical site infections. One patient with prosthetic joint infection continued antimicrobial therapy targeting *C. jeikeium* for more than 90 days, and the other five patients were treated, ranging from 17 to 79 days. Although anti-methicillin-resistant *Staphylococcus aureus* (MRSA) agents, including vancomycin, were the mainstay of treatment. minocycline was used in four cases, including three cases for which it was prescribed as oral step-down therapy. All but one patient, who had a solid tumor with metastases and was treated without removal of the central venous access port, had achieved clinical improvement by Day 14 and was alive at Day 90.

**TABLE 1:** Description of six cases of *Corynebacterium jeikeium* complex infections^*b*^

No.	Strain	Age/sex	Setting	Underlying diseases	Charlson Comorbidity Index	Immunocompromised conditions	History of surgery within 30 days	Diagnosis of infection	Pitt bacteremia score	Sample from which *C. jeikeium* grew	Bacteria grown concomitantly	Antimicrobial treatment targeting *Corynebacterium jeikeium* until Day 90^*a*^	Source control	Clinical improvement by Day 14	Mortality by Day 90
1	TUM 24514	43/M	HA	Retroperitoneal liposarcoma with metastasis	8	Splenectomy	Yes	Postoperative intra-abdominal infection	0	Drained intra-abdominal fluid	*Acinetobacter lwoffii*, *Proteus mirabilis*	Days 0–20: minocycline (PO)	Intra-abdominal abscess drainage	Yes	No
2	TUM 24519	73/M	HA	Pancreatic cancer	1	Splenectomy	Yes	Postoperative intra-abdominal infection	0	Drained intra-abdominal fluid	*Pseudomonas aeruginosa*	**Days 0–7: minocycline (IV**) Days 8–15: amoxicillin (PO) **Days 13–22: minocycline (PO**)	Intra-abdominal abscess drainage	Yes	No
3	TUM 24522	70/M	HA	Rectal cancer	0	None	Yes	Postoperative intra-abdominal infection	0	Drained intra-abdominal fluid	Methicillin-resistant *Staphylococcus aureus*	**Days 4–17: vancomycin (IV**)	Intra-abdominal abscess drainage	Yes	No
4	TUM 24530	53/M	HA	Pancreatic cancer with metastasis	8	None	No	Catheter-related bloodstream infection (central venous port)	0	Blood	None	**Days 4–38: teicoplanin (IV**)	Not performed	No	Yes
5	TUM 24556	59/M	HC	Myxoid liposarcoma with metastasis	8	None	Yes	Vertebral osteomyelitis after spine instrumentation surgery	0	Surgically collected abscess	None	**Days 4–18: vancomycin (IV**) Days 19–79: minocycline (PO)	Surgical debridement	Yes	No
6	TUM 25354	15/M	HA	Osteosarcoma	2	Cellular immunodeficiency	Yes	Prosthetic joint infection after knee arthroplasty	1	Sinus tract discharge	None	**Days 0–17: vancomycin (IV)** **Days 18–31: daptomycin (IV)** **Days 32–42: linezolid (PO)** **Days 43–54: daptomycin (IV**) Days 55–90: minocycline (PO)	Surgical debridement	Yes	No

^*a*^ Antimicrobials to which the *C. jeikeium* complex strains detected were susceptible are shown in bold.

^*b*^ HA, hospital-acquired; HC healthcare-associated.

Although the six strains were identified as *C. jeikeium* by MALDI-TOF MS, all these strains were identified as *C. macclintockiae* by whole-genome sequencing. The ANI of the genomes of these strains was 97.4%–97.9% with the genome of *C. macclintockiae* c9Ua_112^T^ and 87.3%–87.5% with that of *C. jeikeium* NCTC 11915^T^(Table S2).

### Eight-center study of bacteremic strains of *C. jeikeium* complex

While all 33 strains were re-identified as *C. jeikeium* by MALDI Biotyper at the central facility, 32 of these strains were identified as *C. macclintockiae* by whole-genome sequencing (Table S2). The remaining strain (TUM25001) had an ANI with *C. evansiae* c8Ua_174^T^ of 94.6%, which is close to the species identification threshold, and ANIs with *C. jeikeium* NCTC 11915^T^ and *C. macclintockiae* c9Ua_112^T^ of 92.5% and 87.6%, respectively.

### Antimicrobial susceptibility and resistance gene profiles of *C. jeikeium* complex strains

Antimicrobial susceptibility of 39 *C. jeikeium* complex strains collected in the two studies is presented in Table 2 and Table S2. Thirty-four (89.5%) of 38 *C*. *macclintockiae* isolates carried *bla*_Coryne-A_ and were resistant to penicillin. The remaining four strains without *bla*_Coryne-A_ showed relatively low MICs of penicillin, within the intermediate range. Penicillin-resistant strains also had high MICs for ampicillin, and MICs did not decrease with the addition of sulbactam. All strains were also non-susceptible to ceftriaxone, cefepime, and meropenem. Most *C. macclintockiae* strains were resistant to ciprofloxacin, levofloxacin, erythromycin, clindamycin, and trimethoprim-sulfamethoxazole. In line with this observation, most strains had *erm*(X) and had mutations in the QRDRs of *gyrA*.

**TABLE 2:** Antimicrobial susceptibility of 38 *C. macclintockiae* strains collected in this study^*a*,*b*^

	Susceptible, *N* (%)	Intermediate, *N* (%)	Resistant, *N* (%)	MIC_50_ (mg/mL)	MIC_90_ (mg/mL)
Penicillin	0 (0)	4 (10.5)	34 (89.5)	>64	>64
Ampicillin	NA	NA	NA	>64	>64
Ampicillin/sulbactam	NA	NA	NA	>64/32	>64/32
Ceftriaxone	0 (0)	0 (0)	38 (100)	>16	>16
Cefepime	0 (0)	2 (5.3)	36 (94.3)	>16	>16
Meropenem	0 (0)	2 (5.3)	36 (94.3)	>8	>8
Tetracycline	23 (60.5)	1 (2.6)	14 (36.8)	0.5	32
Minocycline	24 (63.2)	8 (21.1)	6 (15.8)	0.12	16
Doxycycline	24 (63.2)	14 (36.8)	0 (0)	0.5	8
Vancomycin	38 (100)	NA	NA	1	1
Teicoplanin	38 (100)	NA	NA	0.5	1
Daptomycin	38 (100)	NA	NA	0.25	0.5
Linezolid	38 (100)	NA	NA	0.5	1
Gentamicin	19 (50)	10 (26.3)	9 (23.7)	4	16
Ciprofloxacin	1 (2.6)	0 (0)	37 (97.4)	>64	>64
Levofloxacin	1 (2.6)	0 (0)	37 (97.4)	>64	>64
Erythromycin	2 (5.3)	0 (0)	36 (94.3)	>16	>16
Clindamycin	2 (5.3)	0 (0)	36 (94.3)	>16	>16
Trimethoprim-sulfamethoxazole	2 (5.3)	NA	36 (94.3)	8/152	>8/152

^*a*^ For teicoplanin, levofloxacin, and minocycline, for which breakpoints are not set in CLSI M45, breakpoints of vancomycin, ciprofloxacin, and doxycycline were alternatively used, respectively.

^*b*^ NA, not applicable.

**Fig 2:**
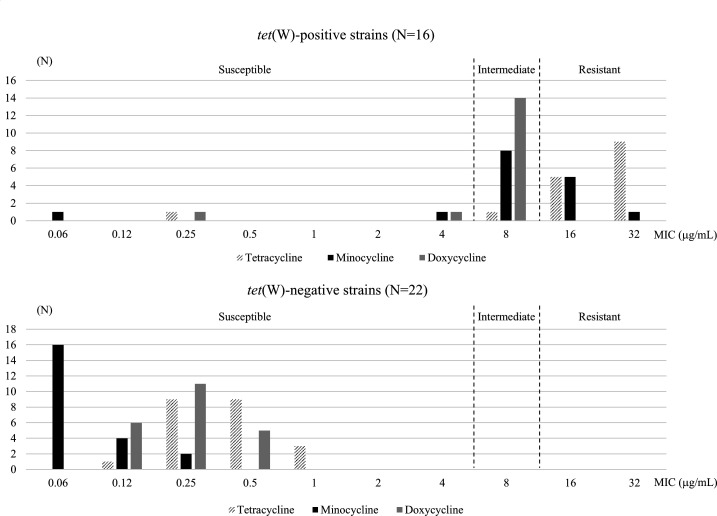
Minimum inhibitory concentrations of *C. macclintockiae* strains with or without *tet*(W) gene for tetracycline derivatives. The breakpoints of tetracycline and doxycycline are indicated by the dotted lines.

Sixteen (42.1%) of 38 *C*. *macclintockiae* strains harbored *tet*(W) gene and were non-susceptible to all tetracycline derivatives except for two strains (TUM24530 and TUM25005) (Fig. 2). The MICs of strains with *tet*(W) were slightly lower for minocycline and doxycycline than for tetracycline, and many strains were intermediate to these drugs. All strains without *tet*(W) were susceptible to all tetracycline derivatives. Nineteen (50%) of 38 *C. macclintockiae* strains were susceptible to gentamicin, and the non-susceptible strains had a higher carriage rate (14/19, 73.7%) of *ant(2″)-Ia*. All *C. macclintockiae* strains were susceptible to all anti-MRSA agents tested. Teicoplanin showed the same or slightly lower MICs than vancomycin.

The *Corynebacterium evansiae* strain TUM25001 had *bla*_Coryne-A_ and a single QRDR mutation of *gyrA*, and was resistant to β-lactams, fluoroquinolones, and trimethoprim-sulfamethoxazole, whereas it was susceptible to erythromycin, clindamycin, gentamicin, tetracycline derivatives, and the anti-MRSA agents (Table S2).

### Phylogenetic analysis of *C. macclintockiae* **strains** in this study

**Fig 3:**
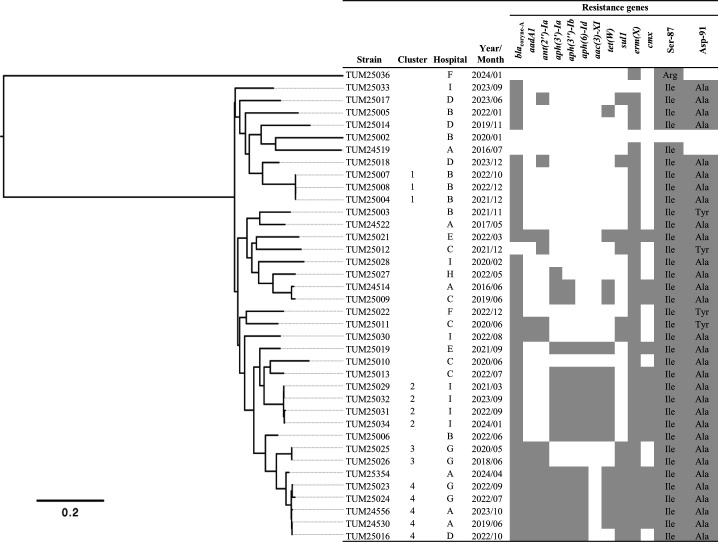
Phylogenetic relationship and resistance gene carriage of *C. macclintockiae* strains. The core-genomic region is 8.4% (203,415 bp/2,410,285 bp) using the genome sequence of *C. macclintockiae* TUM24530 strain as the reference. Resistance gene carriage is indicated by gray boxes. For the quinolone resistance-determining regions of the *gyrA* gene, the amino acids encoded by the mutated genes are documented on the gray boxes.

The *C. macclintockiae* strains collected from nine hospitals in this study were classified into diverse groups by phylogenetic analysis (Fig. 3). In general, genome-wide similarity was consistent with the pattern of resistance gene carriage. An initial phylogenetic analysis identified four clusters that had particularly high similarity. Strains belonging to each of the three clusters (clusters 1–3) were detected in the same hospital, while strains of the remaining one cluster (cluster 4) were detected in three different hospitals.

**Fig 4:**
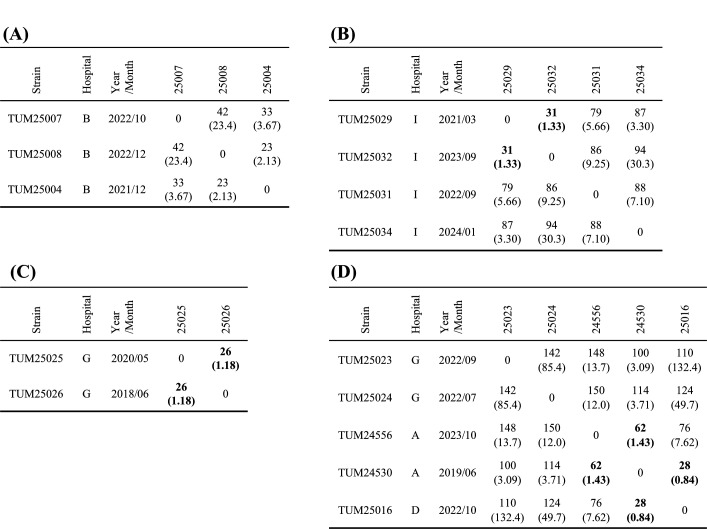
SNP differences among *C. macclintockiae* strains belonging to (**A**) cluster 1, (**B**) cluster 2, (**C**) cluster 3, and (**D**) cluster 4. Genome sequence of *C. macclintockiae* TUM24530 strain (2,410,285 bp) is used as the reference. The core-genomic regions used for identification of SNPs are 89.8% (2,165,369 bp) for cluster 1 strains, 77.5% (1,867,155 bp) for cluster 2 strains, 96.1% (2,315,320 bp) for cluster 3 strains, and 83.1% (2,002,890 bp) for cluster 4 strains. SNP differences between strains are shown in the table, with SNP differences per genome/month in parentheses. Pairs of strains with SNP differences of 2/genome/month or less are shown in bold.

 Core-genome SNP differences between the strains were 23–42 in cluster 1, 31–94 in cluster 2, 26 in cluster 3, and 28–150 in cluster 4 (Fig. 4). The core-genome SNP difference between two blood-derived strains from the same patient isolated 10 months apart performed to estimate the genomic mutation frequency of *C. macclintockiae* was 9, corresponding to 1.04/genome/month. Based on this result and the criteria employed for other bacterial species, we adopted 2 SNPs/genome/month as the conservative threshold for SNP differences between strains that possibly originated from the same clone (21, 22). There were four strain pairs with SNP differences that fell within this range, three of which were strains detected at the same hospital (Fig. 4).

### Molecular characteristics of global *C. jeikeium* complex strains

**Fig 5:**
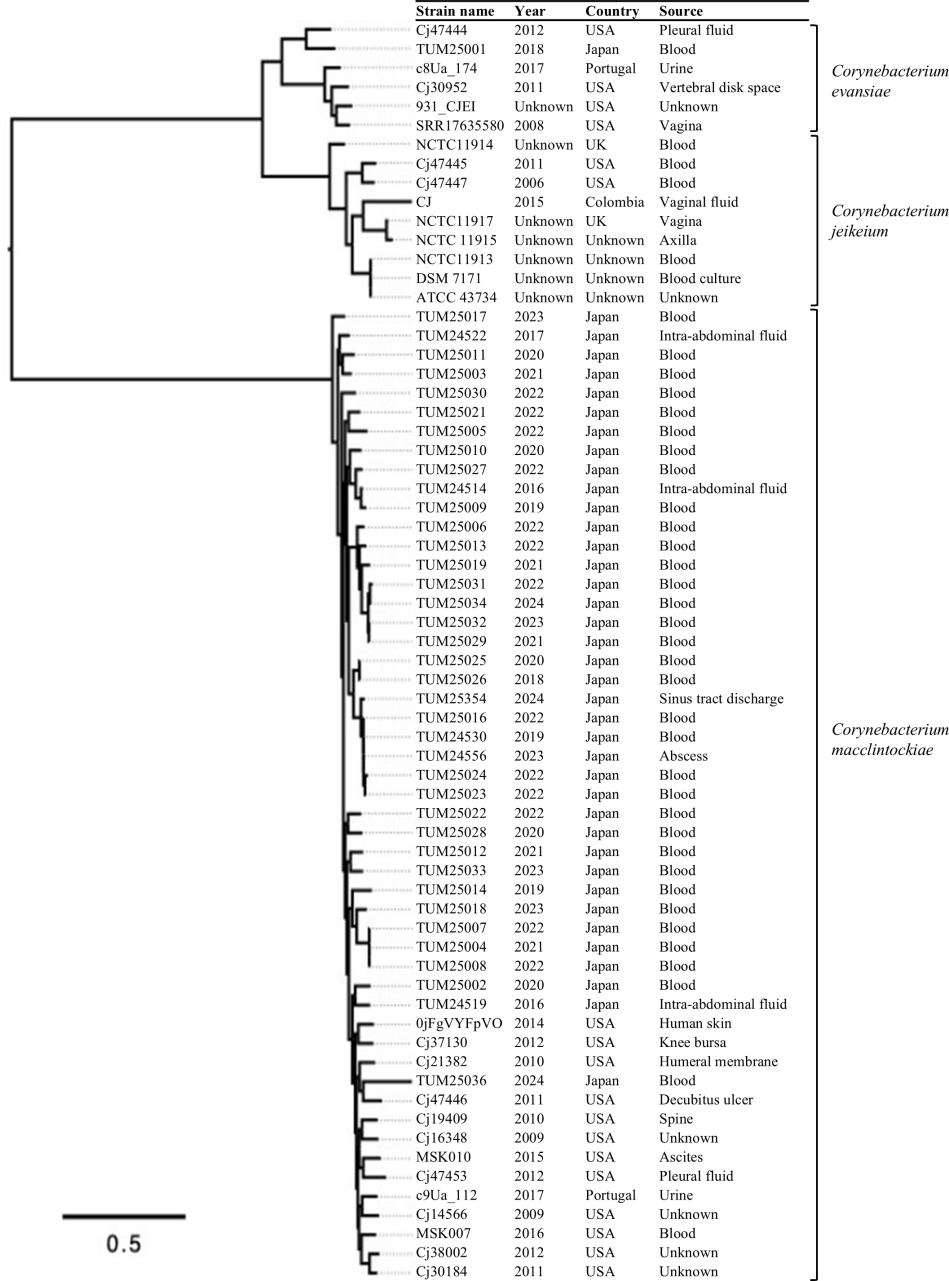
Phylogenetic analysis of global *C. jeikeium* complex strains. The core-genomic region is 9.1% (220,116 bp/2,410,285 bp) using the genome sequence of *C. macclintockiae* TUM24530 strain as the reference. Information on strains using genomic data in GenBank was adopted from the GenBank registries and relevant literatures (23). Although the ANI of two strains (Cj47444 and TUM25001) and the type strain of *C. evansiae* was slightly below the threshold (94.9% and 94.6%), these strains were tentatively regarded as *C. evansiae* because there were no other type strains with average nucleotide identity of 95% or higher.

The core-genome SNP analysis was performed on 39 *C*. *jeikeium* complex strains collected in our study and 27 *C*. *jeikeium* complex strains for which the genomic data were registered in GenBank (Table S3; Fig. 5). Of the strains registered in GenBank, 18 were from the United States, two each from the United Kingdom and Portugal, and one from Colombia. While six of the GenBank-registered strains were of unknown sample origin, all other 21 strains were derived from human specimens with 14 strains from sterile site cultures (e.g., blood, body cavity fluid, osteoarticular specimen). Genetic identification identified 51 isolates as *C. macclintockiae*, 9 as *C. jeikeium*, and 6 as *C. evansiae*.

**Fig 6:**
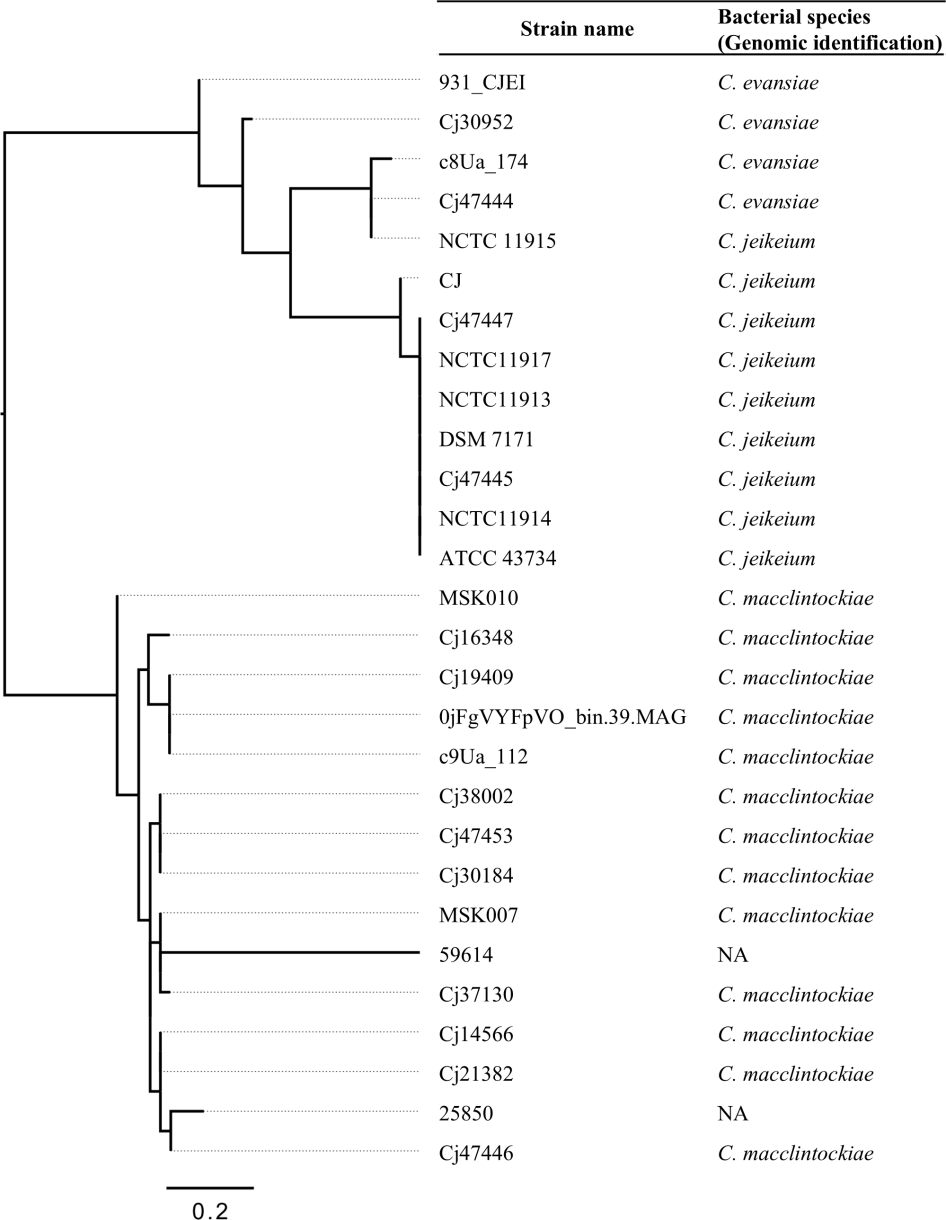
Phylogenetic analysis of 16S rRNA and *rpoB* sequence of global *C. jeikeium* complex strains. NA, not applicable.

 A dendrogram based on 16S rRNA and *rpoB* sequence similarity of strains 25850 and 59614, and *C. jeikeium* complex strains for which genome sequences were registered in GenBank showed that these two strains formed a single cluster with other *C. macclintockiae* strains, suggesting that they are *C. macclintockiae* (Fig. 6).

## DISCUSSION

This study demonstrated that most strains causing infections in Japan and identified as *C. jeikeium* by MALDI-TOF MS were *C. macclintockiae* based on the genomic analysis. Although *C. macclintockiae* was generally multidrug-resistant, reflecting carriage of relevant antimicrobial resistance genes, possible therapeutic options such as teicoplanin and tetracycline derivatives were recognized. Additional analyses using genomic data registered in public databases suggest that *C. macclintockiae* is of global clinical importance among *C. jeikeium* complex, consisting of *C. jeikeium sensu stricto*, *Corynebacterium macclintockiae*, and *Corynebacterium evansiae*.

 Although patients with hematologic malignancies are well known to be susceptible to *C. jeikeium* infection, our single-center study documented six cases of infection in patients without hematologic malignancies. Infective endocarditis and osteoarticular infections caused by *C. jeikeium*in patients without hematologic malignancies have been reported repeatedly (24–26). These infections occur primarily in patients with prostheses, as observed in two cases with prosthetic osteoarticular infections in our study. Additionally, three cases of postoperative intra-abdominal infection and a case of catheter-related bloodstream infection were observed. The increased frequency of identification of the *C. jeikeium* complex with the widespread use of MALDI-TOF MS may provide evidence of *C. jeikeium* complex infections in previously unrecognized hosts and organs (27).

Of the *C. jeikeium* complex strains collected in this study, all except one were genomically identified as *C. macclintockiae. C. macclintockiae* was reported in 2023 as a newly identified species by whole-genome sequencing analysis of an isolate detected from the urine of a healthy woman (28). The clinical importance of this species was highlighted in the current study, with all strains being causative organisms of infections or detected in multiple sets of blood culture. Thirteen *C. jeikeium* complex clinical isolates collected in the United States were previously classified into four genomospecies by whole-genome sequencing analysis (23). Nine of these strains were identified as *C. macclintockiae* in the analysis using registered genomic data in the present study (Fig. 5). In fact, the genetic diversity within *C. jeikeium* strains was noted in an analysis conducted 30 years ago using DNA-DNA hybridization, and the recent widespread use of bacterial genome analysis has made this point even clearer (29). Analysis of 16S rRNA and *rpoB* sequences of clinical strains in France also suggested the presence of *C. macclintockiae* among *C. jeikeium* complex strains (Fig. 6). Although these findings suggest that *C. macclintockiae* is involved in human infections at a certain frequency outside of Japan, detailed global distribution of *C. macclintockiae* must await additional analysis in the future, since only a limited number of strains, mainly from Japan, have been analyzed. Phylogenetic analysis suggested that the genetic backgrounds of the *C. macclintockiae* strains reported from the United States and Japan were different, but the limited number of strains analyzed and differences in collection periods preclude conclusions.

Similar to the observations using MALDI Biotyper in this study, it was reported that the type strains of *C. macclintockiae* and *C. evansiae* were misidentified as *C. jeikeium* using Vitek MS, another MALDI-TOF MS bacterial identification system (28). Given that identification methods generally available in microbiology laboratories at present, including MALDI-TOF MS, cannot accurately distinguish the three species in the *C. jeikeium* complex, it would be appropriate to collectively report as “*Corynebacterium jeikeium* complex” when bacteria detected in a clinical specimen are identified as one of these three species.

 SNP analysis identified four clusters of *C. macclintockiae* strains with high genomic similarity (Fig. 3 and 4). Strains belonging to each of the three clusters (clusters 1–3) were detected only in one hospital, suggesting nosocomial transmission. Indeed, two strain pairs in these clusters had SNP differences small enough to support the same origin of the strains. Several studies have reported nosocomial outbreaks of *C. striatum*, including one in which multiple clusters were spread across hospital wards (30–32). Our results suggest that a similar situation may be occurring in the *C. jeikeium* complex. The increased recognition of *Corynebacterium* spp. as human pathogens in recent years and the resulting increased frequency of performing species identification and antimicrobial susceptibility testing on these pathogens may lead to recognition of more cases of nosocomial transmission of *C. jeikeium* complex in the future. Interestingly, strains belonging to cluster 4, which harbored many resistance genes, were detected in three geographically separated hospitals, suggesting that it may be an epidemiologically important cluster.

 Overall, *C. macclintockiae* strains analyzed in this study were multidrug-resistant. As expected, the genome-wide similarity and antimicrobial resistance gene profiles of the strains were consistent. Since complete genome analysis using long-read sequencing was not performed in this study, except for one strain used as a reference strain of core-genome SNP analysis, information on the genomic location of the resistance genes is unclear. Future application of complete genome analysis to a wide range of *C. macclintockiae* strains would provide information on the genetic environment of the resistance genes and on the spread of resistance genes between different clones via conjugative plasmids.

Antimicrobial susceptibility of several antimicrobials deserves special mention from two perspectives: the need for alternatives in vancomycin-intolerant patients and for oral treatment options in patients requiring long-term treatment. Teicoplanin showed the same or slightly lower MICs than vancomycin in all strains and was used in one case as in a previous report (33). In a retrospective study comparing teicoplanin and vancomycin for the treatment of glycopeptide-susceptible *Enterococcus faecium* bacteremia, teicoplanin showed therapeutic effectiveness that was comparable to vancomycin, with a lower incidence of acute kidney injury (34). Patients with *C. jeikeium* complex infections are often elderly or have underlying medical conditions with a high risk for renal impairment. Further studies are needed to verify the clinical effectiveness of teicoplanin for *C. jeikeium* complex infections, and breakpoints should be established. Although all strains in this study were susceptible to daptomycin, there is concern about resistance development after drug exposure, as shown *in vitro* studies and has been reported at high frequency in patients with *C. striatum* infections treated with daptomycin (35–37). Linezolid showed favorable antimicrobial susceptibility in this study, as in previous studies (2). Therefore, this drug is a treatment option for *C. jeikeium* complex infections, but long-term use may be hampered by side effects such as hematological toxicity (38). Tetracycline derivatives, doxycycline, and minocycline have been used as oral suppressive antimicrobial agents in previous reports of osteoarticular infections due to *Corynebacterium* spp. (26). Minocycline is available orally in Japan and was used for treatment in four cases in this study. About 60% (22/38) of the *C. macclintockiae* strains lacked *tet*(W) and showed MICs substantially lower than the susceptibility breakpoints for all tetracycline derivatives. On the other hand, strains with *tet*(W) exhibited MICs in the intermediate range or lowest within the resistant range for doxycycline and minocycline. Standardized susceptibility testing should be performed to avoid the use of tetracycline derivatives in the treatment of non-susceptible strains, as was observed in three cases in this study. Clinical validation of the current doxycycline breakpoints and establishment of breakpoints for minocycline are also necessary.

This study has several limitations. First, the number of cases with clinical information was limited. Nevertheless, even with a small number of cases, a variety of infections caused by *C. macclintockiae*, including those requiring long-term antimicrobial therapy, were included. Second, the source of bacteremia in the cases in the eight-center study is unknown. However, only cases in which *C. jeikeium* complex was detected in multiple sets of blood cultures were included, which strongly suggests that they were true bacteremia cases. Third, the validity of the SNP difference threshold used to identify possible clonal transmission in this study is uncertain. The fact that three of the four strain pairs identified by the applied threshold were strain pairs detected in the same hospital supported the validity of the threshold, but the remaining one was a strain pair detected in geographically distant hospitals. Further accumulation of genomic data of clinical isolates of *C. jeikeium* complex worldwide would identify a reasonable threshold. Fourth, the route and extent of nosocomial transmission of *C. macclintockiae* isolates are unknown because hospital epidemiological information was not obtained.

In conclusion, the study demonstrates the clinical importance of *C. macclintockiae*, one of the genomospecies of *C. jeikeium* complex. *C. macclintockiae* causes a variety of infections and is multidrug-resistant, necessitating exploration of antimicrobial treatment options. Further accumulation of information is needed to determine whether *C. macclintockiae* differs from other genomospecies of *C. jeikeium* complex in terms of natural habitat, routes, and characteristics of human infection.

## Data Availability

Draft genome sequences have been deposited in the NCBI database under BioProject accession number PRJNA1219363. The complete genome sequence of one strain (TUM24530) was registered in the NCBI database under BioProject accession number PRJNA1219775.

## SUPPLEMENTAL MATERIAL

Table S1 jcm.00500-25-s0001.xlsx Definition of immunosuppression in this study.

Table S2 jcm.00500-25-s0002.xlsx *Corynebacterium jeikeium* complex strains collected in this study.

Table S3 jcm.00500-25-s0003.xlsx Whole-genome sequencing analysis data downloaded from GenBank for comparative genome analysis.

ASM does not own the copyrights to Supplemental Material that may be linked to, or accessed through, an article. The authors have granted ASM a non-exclusive, world-wide license to publish the Supplemental Material files. Please contact the corresponding author directly for reuse.
